# Anthropometric study of hip with computed tomography scan

**DOI:** 10.4103/0019-5413.65135

**Published:** 2010

**Authors:** Aditya V Maheshwari

**Affiliations:** *Department of Orthopedics, Washington Hospital Center, 110 Irving ST NW, Washington DC, USA-200 10*

Sir,

I read with great interest the manuscript by Saikia *et al*. ‘Anthropometric study of the hip joint in North-Eastern region population with computed tomography scan”.^1^ I must congratulate the authors for this study. Although there is enough Western literature on this topic, Indian literature is sparse. Thus this data warrants attention considering the fact that more than 1/6^th^ of the world’s population is from the Indian origin.

However, I would like to draw attention to two particular values; femoral anteversion (FNA) and the femoral neck shaft angles (FNS). The mean FNA in this study was 20.4° (8°-40°) and the mean FNS was 139.5° (118°-150°). Although the mean may not be the best values to compare, the median values are also higher (20° and 140°). Thus, both FNA and FNS in this study appear pathologic when compared to normal values on various Caucasian and Mongoloids races as well as on Indian subjects.^2^–^10^ These studies have shown the normal mean FNA as 8-15° with a normal FNS of 123-135°. In several previous studies,^3^–^7^ the mean FNA was around 8°, whereas this was the minimum value in the study by Saikia *et al*. In our previous unpublished CT study (Indian Orthopaedic Association Conference (IOACON) 2002; Agra, India), the mean FNS in normal Indian adults was 130°. More than 80% of the cases had FNS less than 135° and only 2.5% of the cases had FNS over 140°. Similarly, in another Indian study the maximum FNS was 140°.^8^

The mean acetabular anteversion (AA) in this study (18.2°) was comparable to the Western studies^5^,^10^ and to 19° in our previous unpublished CT study on normal Indian adults (IOACON 2002; Agra, India). Thus increased FNA would create incongruence at the hip leading to uneven joint force reactions and ultimately leading to instability and/or accelerated osteoarthritis (OA).^10^ However, to the best of our knowledge, the incidence of developmental dysplasia of the hip (DDH) and/or primary OA of the hip has not been reported to be higher in the North Eastern population as compared to the rest of India or the world. Apart from the consideration of implant design, the other implication of increased FNA would be during cup implantation in a non-cemented total hip arthroplasty, where the cup needs to be put in less anteversion to be in a ‘safe zone’ of combined anteversion for increased stability and reduced wear.^11^

We agree that racial and geographic variations do exist in proximal femoral morphology. But it is still difficult to accept these higher values as normal. One explanation of these higher values may be the authors’ use of Murphy *et al*. method,^12^ which uses the center of the head rather than the center of the neck for estimation of the FNA. Several studies have shown that majority of the femoral heads are not in the center of the femoral neck.^3^,^7^,^12^,^13^ In spite of all these possible explanations, the findings of this study 1 still show an incomprehensible difference, not only when compared with international literature but also with other Indian studies.
